# New Chemically Amplified Positive Photoresist with Phenolic Resin Modified by GMA and BOC Protection

**DOI:** 10.3390/polym15071598

**Published:** 2023-03-23

**Authors:** Junjun Liu, Wenbing Kang

**Affiliations:** National Engineering Research Center for Colloidal Materials, School of Chemistry and Chemical Engineering, Shandong University, Jinan 250100, China

**Keywords:** phenolic resin epoxy modification, BOC protection, chemically amplified photoresist, UV exposure

## Abstract

In this paper, a chemically amplified (CA) i-line photoresist system is described including a phenolic resin modified with glycidyl methacrylate (GMA) addition and protected with di-tert-butyl dicarbonate (BOC group), here called JB resin. JB resin with different degrees of BOC protection was synthesized and characterized with ultraviolet spectrophotometry, Fourier transform infrared spectroscopy and gel permeation chromatography. These resins were also evaluated in CA resists by formulating the JB resin with a photoacid generator (PAG) and tested at 405 nm and 365 nm exposure wavelengths. The BOC protection ratio at approximately 25 mol% of the Novolak phenol group showed the best performance. The resist showed high sensitivity (approximately 190 mJ/cm^2^), high resolution and good alkali developer resistance with reliable repeatability, indicating the great practical potential of this JB resist system.

## 1. Introduction

The photoresist is an essential product for semiconductor manufacturing and chip packaging. It also has various applications in other industry areas for image making [1,2]. Photoresists are categorized by exposure light wavelength and imaging type (positive or negative). Normally, they contain polymer resins as the main components, and other photosensitive components. In the field of photoresist materials, the “Novolak/DNQ” photoresist with phenolic resin and diazonaphthoquinone ester (a photoactive compound, PAC) as the main components is widely used in the g-line (436 nm), h-line (405 nm) and i-line (365 nm) light and with a mixture of g-, h- and i-line light. The DNQ photoresist utilizes the interaction of phenolic resin and DNQ to make the film less soluble in the standard 2.38 wt% tetramethyl ammonium hydroxide (TMAH) developer. Exposure to light decomposes DNQ, and the inhibitory effect is removed. Then, the solubility of the exposed film area in TMAH is greatly enhanced. Various techniques have been used to adjust the performance of the Novolak/DNQ resist system by using resin modification (phenol types, molecular weight) and DNQ types and amount, including additives to reduce or enhance the TMAH solubility of the resist film. There are many efforts to improve such i-line-type photoresists due to their importance in industry. The modification of resins in recent years has mainly focused on heat resistance [3,4], rheology, adhesion, stability, thermal decomposition temperature properties [5] and mechanical properties. These include aromatic amines and aromatic compounds [6,7,8], silicones, rosins, epoxy resins, furans [9], tannins [10], lignin [11], boron and molybdenum. Researchers have explored different performance aspects of phenolic resins by such modifications. Y. Chen et al. [12] used the carbon–carbon unsaturated bond of the maleimide ring to react with the active hydrogen on the phenolic hydroxyl group to improve the heat resistance of modified phenolic resins. M. Gao et al. [13] chose dicyandiamide as a modifier and added it to the reaction vessel at the same time as the raw material to prepare dicyandiamide with excellent mechanical and flame-retardant properties under alkaline conditions. F. Wang et al. [14] used the principle that organosilicon modifiers can react with phenolic hydroxyl groups to modify Novolak resins to realize applications in image flaw display and separation of mixed signals. H. Kimura et al. [15] investigated the curing reaction of bisphenol A-based benzoxazine (Ba) with epoxy resin (Ep) using potential curing agents, and it was demonstrated that cured resins containing Ba and Ep with potential curing agents exhibited good heat resistance, flame retardancy, mechanical properties and electrical insulation properties. Research on Novolak resins for photoresists has focused on the modification of Novolak resins in terms of diazo compounds, azide compounds and epoxy resins. Modified Novolak resins have also achieved lithographic performance in different aspects [16,17,18]. However, there is still a need for i-line photoresists to have higher sensitivity and resolution than traditional DNQ-based resists.

Chemically amplified resist systems [19,20] (CA resists) in DUV (248 nm), ArF (193 nm) and EUV (13.5 nm) use partial protection of the alkaline developer soluble group in the resin (mainly using BOC and/or acetal groups) and deprotect the group by exposure to generate strong acid, then postexposure baking (PEB) to deprotect the group, making the resin fully soluble in TMAH. We have explored introducing the CA-type resist system to i-line for this purpose. We found that better performance could be achieved by the reaction of epoxy-type materials such as glycidyl methacrylate (GMA) with the phenolic resin and then the partial protection of the remaining phenolic hydroxyl group by the BOC group. Normal phenolic resin has very high solubility in the TMAH developer. The alkali dissolution rate (ADR) of resin depends on the phenolic components, molecular weight and synthesis method and is difficult to control. The phenolic/DNQ resist system utilizes resin and PAC, including additives, to control the final resist performance. However, in a CA-type resist, the main components are an active group-protected resin and a photoacid generator. The resin itself will in great part define the resist performance. We have to protect the essential part of the phenolic phenol group to make it insoluble in TMAH. Introducing a BOC protection group into a poly-(p-hydroxystyrene) resin by the reaction with di-tert-butyl dicarbonate is a traditional method for chemically amplified resists. This BOC group could be deprotected in the presence of a strong acid at the heating temperature. Phenolic resin has wide application and is used in very large-scale production. It is a major component in Novolak/DNQ resists for display and IC manufacturing areas and has a very good cost advantage. We chose phenolic resin as a potential resin for i-line CA-type resist-based resins. Thus, we studied the protection of phenol groups with BOC groups at different ratios. The BOC protection ratio needs to be optimized for different phenolic resins to obtain sufficient TMAH developer resistivity. We used an i-line CA resist formulation containing i-line photoactive PAG to screen the performance of the modified resin. Our results showed that BOC protection only in the phenolic resin system could not give better CA resistance performance. However, the resin with glycidyl methacrylate (GMA) adduction to the phenol hydroxyl group at a certain degree and a proper BOC protection ratio showed better performance and a wide window.

**Figure 1:**
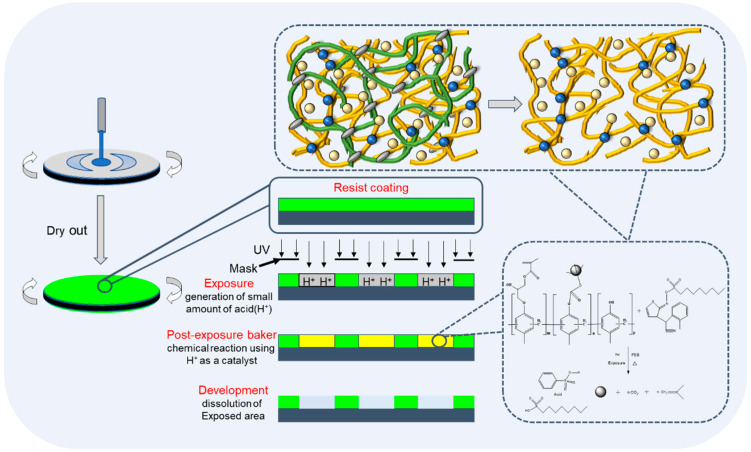
Lithography schematic diagram of JB series photoresist.

**Figure 2:**
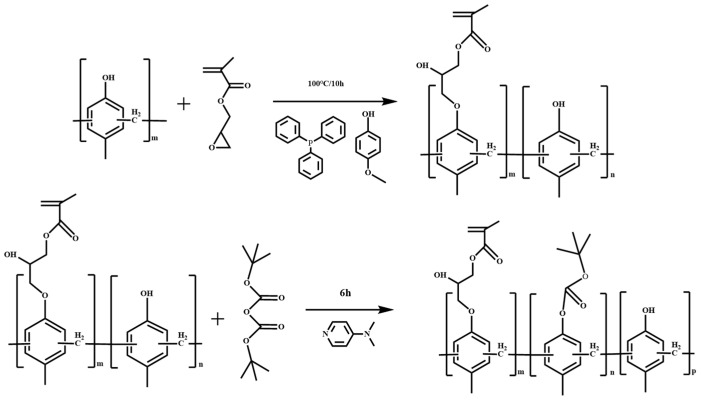
Synthetic scheme of JB.

Normally, developer solubility is adjusted by changing the composition, molecular weight and reaction conditions. It is used with DNQ to control the ADR and dissolution performance after light exposure. The phenolic resin is modified by introducing an epoxy group (epoxy Novolak for crosslinking-type coating and negative photoresist such as SU-8) and the epoxy ring opening by the phenol group to form a side ether/alcohol group. Glycidyl methacrylate modifies polymers in three ways: epoxy groups in branched chains, epoxy ring opening and reactions of compounds with active hydrogen [21,22]. In this work, the phenolic resin is modified by means of a ring opening etherification reaction between the epoxy group of GMA and the phenolic hydroxyl group, forming a phenolic resin with a side group with alkyl chain-linked acrylate. This improved the resin’s solubility and it can be used as a photopolymerizable polymer in coatings and negative photoresist systems [23]. Our study showed that phenolic resin with a GMA side group and BOC protection showed good performance as a CA-type positive resist, providing a low-cost material with a good supply ability. The lithography schematic diagram of the JB (phenolic resin containing glycidyl methacrylate-adducted side group and protected with di-tert-butyl dicarbonate was named JB) series photoresist is shown in Figure 1. The synthetic scheme of JB is shown in Figure 2.

The CA-type positive photoresist formulation evaluation in this work was carried out in propylene glycol methyl ether acetate (PGMEA) as the solvent and PAG-like octylsulfonyl ester (HG-108). Several resins with different BOC protection ratios were tested in CA-positive resists. It was found that the synthetic resin with a BOC ratio of 25 mol% (JB25) had good adhesion and coating on the silicon substrate, which resulted in good developer resistance (less film loss) and good resolution. The JB25 sample achieved excellent resolution with a 4 μm mask. As a result, partial blocking of the phenolic hydroxyl group with GMA and by BOC protection for the chemically amplified positive resist was confirmed as a good approach. Our work provides a new idea to utilize industrially available and low-cost phenolic resin in CA-positive resists by applying GMA and BOC protection. This opened a new route for i-line- and g-line-type CA photoresists for IC, FPD and chip packaging applications.

## 2. Materials and Methods

### 2.1. Raw Materials

The raw materials used for the experiments included phenolic resin purchased from Beijing Technology (Shandong, China) Co., Ltd. Glycidyl methacrylate (AR, 97%, containing 100 ppm MEHQ stabilizer), propylene glycol methyl ether acetate (AR, 99%, containing 50 ppm BHT stabilizer), triphenylphosphine (AR, 99%), di-tert-butyl dicarbonate (AR, 98%) and 4-dimethylaminopyridine (AR, 99%) were purchased from Shanghai Maclean Biochemical Technology Co., Ltd. (Shanghai, China). p-Methyl phenol (AR, 99%) was purchased from Shanghai Dipper Biotechnology Co., Ltd. (Shanghai, China).

### 2.2. Synthesis of Materials

JB resins with different BOC protection ratios were prepared according to the following procedure:

(1) Addition reaction of GMA (17.1 g, 15 mol%) to phenolic resin JB480 (82.28 g, 100 mol%) in PGMEA (234. 2 g) as the solvent in a round flask with magnetic stirring. First, the JB480 (82.28 g) resin was mixed into PGMEA (204.2 g) and stirred at approximately 60 °C until the resin was completely dissolved, and then the solution was cooled to approximately 35 °C. The inhibitor p-methoxyphenol was added into the solution at approximately 50 ppm. Then, the required amount of GMA (17.1 g) was added into the reaction flask, and the temperature was raised to 70 °C. Finally, a solution of triphenylphosphine (0.98 g) in PGMEA (30 g) was added dropwise to the reaction solution through a dropping funnel. The reaction was continued at approximately 100°C for 10 h to give GMA-adducted resin solution JB00.
(2) BOC protection: Based on the BOC protection ratio of resin JB480, the molar ratios of JB480 and BOC were 100:20, 100:25 and 100:30 molar, hereinafter referred to as JB20, JB25 and JB30, respectively. DMAP (the quantities of DMAP of 0.15 g, 0.19 g and 0.22 g correspond to JB20, JB25 and JB30, respectively) was dissolved into 5 g of PGMEA and then slowly added dropwise to a solution of JB00 (167.2 g) with stirring at room temperature. The BOC reagent (the mass and molar ratio of BOC to be added to JB20, JB25 and JB30 were 14.8 g, 18.6 g, 22.3 g and 20 mol%, 25 mol%, 30 mol%, respectively) in 5 g of PGMEA was added to the reaction solution. The mixture was stirred with a magnet stirrer at 25 °C for 6 h. The resulting BOC-protected synthetic JB resin was filtered with a PTFE membrane filter for further study. The synthesis process of JB20, JB25 and JB30 was consistent.

### 2.3. Material Characterization

Scanning electron microscopy (SEM, S-4800, Hitachi of Japan, Tokyo, Japan) was used to study the morphology and structure of the samples, and it was performed with an electron voltage of 5 kV through an SE2 lens. Fourier transform infrared spectroscopy (FT-IR, Tensor II, Bruker, Billerica, MA, USA) was used to study the surface structure of the samples. The samples were applied dropwise on potassium bromide tablets, baked dry and tested. The wavelength range was 500–4000 cm^−1^ at room temperature. The absorption properties of the modified resins were characterized with ultraviolet–visible spectra (UV–Vis, Carry5000, Agilent, Palo Alto, CA, USA). UV–Vis spectra were recorded with a Hitachi U-4100 UV–Vis spectrophotometer, and the specification wavelength range was 400–4000 nm. The test concentration was 0.06 mg/mL. The relative molecular weight and relative molecular number distributions of the polymers were measured by using gel permeation chromatography (GPC, BI-MWA, Brookhaven Instruments Corporation, Holtsville, NY, USA), in which the measurement range of Mw was from 500 to 109 Daltons, and the mobile phase was DMF at a flow rate of 1.0 mL/min. The film thickness was determined using an F20 Thin-Film Analyzer (Filmetrics, San Diego, CA, USA), and the measured film thickness range was 10 nm–100 μm. A contact angle tester (DSA25, KRUSS, Hamburg, Germany) was used to measure the static and rolling contact angles of pure water on the material. The contact angle tester measured the angle range of 1°–180° by using the seat-drop method, and the experiment was carried out with 6.4 multiplier optical zoom, 10 pictures per second and 0.01 mN/m resolution.

### 2.4. Lithography Evaluation

JB20, JB25 and JB30 photoresist samples were obtained by adding 2 wt% of HG-108 photoacid generator to the synthesized JB resin material solution in PGMEA and filtered through a 0.2 μm PTFE membrane filter. The photoresists were then spin coated onto silicon wafers by static dispensing and baked with a hot plate at approximately 110 °C for 3 min. All samples were subjected to UV exposure with an LED light (wavelength of 365 nm) source through a chrome mask with an exposure time of 15 s (exposure energy of approximately 190 mJ/cm^2^). Then, postexposure baking was applied to the wafer, and it was developed with 2.38 wt% tetramethyl ammonium hydroxide aqueous solution (TMAH) for 90 s at 23 °C and rinsed with water.

## 3. Results and Discussion

### 3.1. Chemical Structure and Molecular Properties

**Figure 3:**
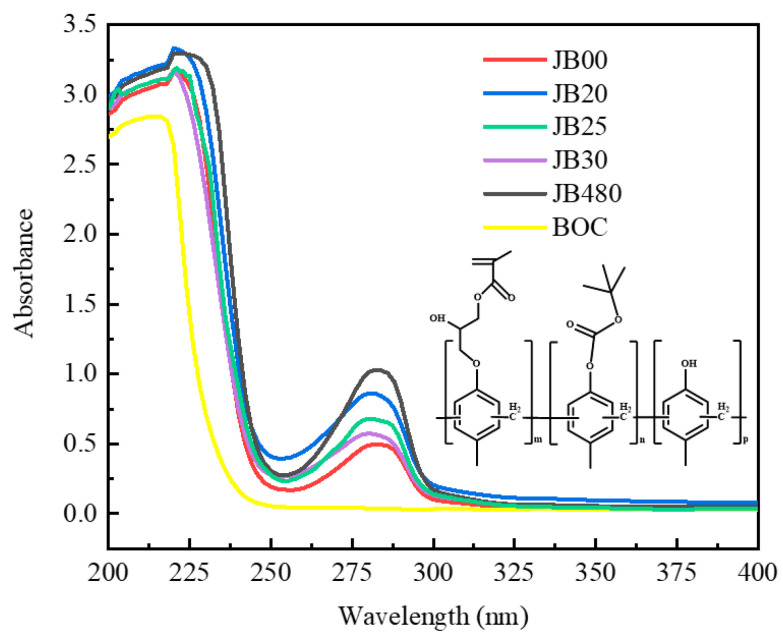
UV spectra of JB00, JB20, JB25, JB30, JB and BOC.

It can be seen from the UV spectrum that JB20, JB25 and JB30 have strong absorption peaks at 220 nm and 280 nm. The electronic absorption band of the carbonyl chromophores in BOC is at 195 nm. The redshift in absorbance is due to the combination of the hydroxyl group of JB20, JB25 and JB30 with a BOC group containing π-bonds (p-electron), resulting in a conjugation effect, and the group generally has an electron-pushing conjugate effect. The substituents on the benzene rings of JB20, JB25 and JB30 shift the absorption peaks in the long wavelength direction and enhance the absorbance, and the magnitude of the absorption peak shift due to the substituents is affected by the possible tautomerism in the molecule. The hydroxyl group acts as an electron-donating group and enhances the chromogenic group at the same time. Even if the benzene ring is connected with an electron-withdrawing group, the maximum absorption wavelength of the substituted benzene will also show a certain additive effect; thus, the modification of BOC has an effect on the UV absorbance of polymers JB20, JB25 and JB30. The strong absorption of JB20, JB25 and JB30 at 204 nm indicates the presence of benzene in aromatic compounds in the E2-band. The strong absorption of JB20, JB25 and JB30 at 210-250 nm indicates a π-bonded conjugated K absorption band. The weak absorption of JB20, JB25 and JB30 in the R-band (the R-band is the result of the transition of the lone pair electrons of the unbonded electrons on the heteroatom connected to the double bond to the π* antibonding orbital, which can be simply expressed as n→π*) at 250-300 nm is because the lone pair of electrons on the unbonded C=O attached to the double bond jumps to the π* reverse orbital. In addition, the molecule also contains a simple nonconjugated and n-electron carbonyl chromogenic group [24]. It is seen from Figure 3 that JB20, JB25 and JB30 are almost transparent near 250 nm and above 300 nm, so there is no conjugated system in the molecules of JB20, JB25 and JB30 in the band above 300 nm.

**Figure 4:**
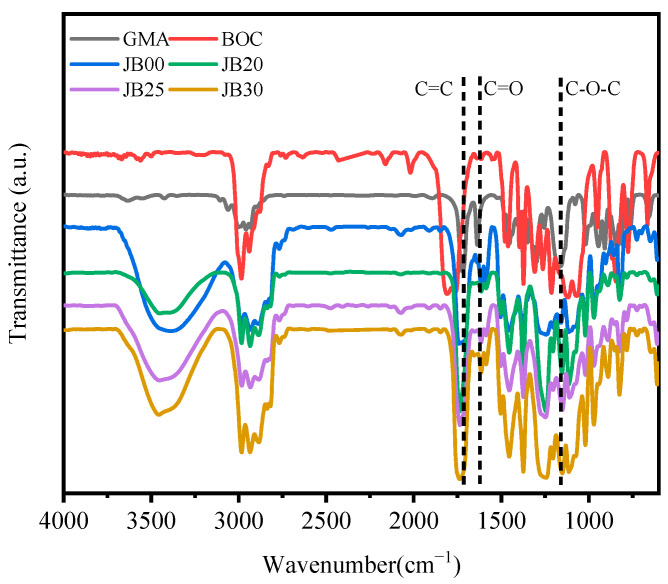
Infrared spectra of GMA, BOC, JB00, JB20, JB25 and JB30.

The infrared spectra of JB00, JB20, JB25 and JB30 with different BOC contents are shown in Figure 4. The peaks at approximately 1735 and 1635 cm^−1^ correspond to the C=C and C=O stretching vibrations, respectively [25,26]. The presence of a double split peak at 1165 cm^−1^ proves the existence of the methacrylate GMA double bond, and the peak at 1165 cm^−1^ corresponds to the symmetric stretching resonance of the other C-O-C group [27]. JB00, JB20, JB25 and JB30 have no obvious sharp peak shape at 1165 cm^−1^, but JB00, JB20, JB25 and JB30 correspond to a double splitting asymmetric stretching resonance of C-O-C at 1242 cm^−1^, proving the ring opening of the epoxy group of GMA. GMA corresponds to the stretching resonance of carbonyl C=O at 1716 cm^−1^, while JB00, JB20, JB25 and JB30 all correspond to the strong absorption peak of carbonyl C=O at 1735 cm^−1^, which proves the partial ring opening reaction of the epoxy group of GMA grafted onto JB480. Peaks of BOC at 1795 cm^−1^ and 1117 cm^−1^ correspond to the strong absorption peaks of carbonyl C=O and C-O, respectively, and JB20, JB25 and JB30 correspond to the stretching resonance of carbonyl C=O at 1739, 1748, 1735 and 1744 cm^−1^, respectively. The absence or a significant decrease in the carbonyl signal can be attributed to the relatively stable lone pair of electrons of carbonyl oxygen [28]. The absence of absorption peaks corresponding to C-O in JB00, JB20, JB25 and JB30 proves that BOC successfully reacted with JB00 by grafting, corresponding to the formation of JB20, JB25 and JB30.

**Figure 5:**
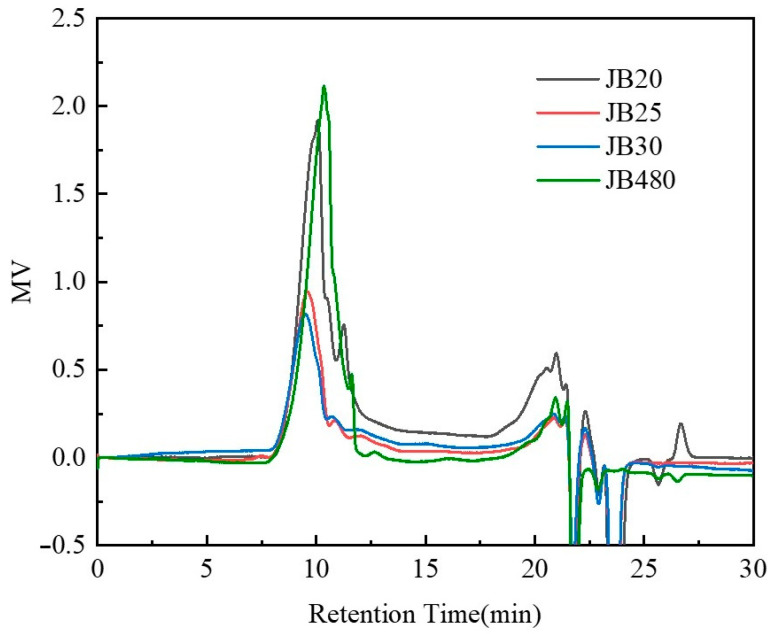
GPC results of the JB samples.

The JB resins were analyzed with gel permeation chromatography (GPC) using polystyrene as the standard and tetrahydrofuran as the eluent. The results are summarized in Table 1 and graphed in Figure 5. According to Figure 5, the peaks of the three curves from 7–15 min are the main polymer peaks, and the peaks after 17 min are mainly reaction and eluting solvents. After GMA and BOC protection, the Mw decreased and the polydispersity also changed. However, there are small differences between samples with different BOC ratios. This may be due to the modification of JB480 with GMA and BOC changing the morphology of original phenolic resin.

### 3.2. Quality Evaluation of the Modified Phenolic Resins

In this work, different GMA contents (0 mol%, 5 mol%, 10 mol%, 15 mol%, 20 mol%, 25 mol%, 30 mol%) were designed to investigate the variation in GMA on the grafting of phenolic resins. As shown in Table 2, the variation in film thickness of phenolic resin grafted with different proportions of GMA shows that the film thickness of the synthesized resin changed with increasing proportion of GMA, and its film thickness loss value basically showed a decreasing trend. This could indicate the successful grafting of GMA and that GMA was consumed and transformed during the reaction process. The GMA proportion of 15 mol% is a relatively suitable choice. This choice is not only related to alkali resistance, but also takes into account various influencing factors, such as individual and compound modifications.

**Figure 6:**
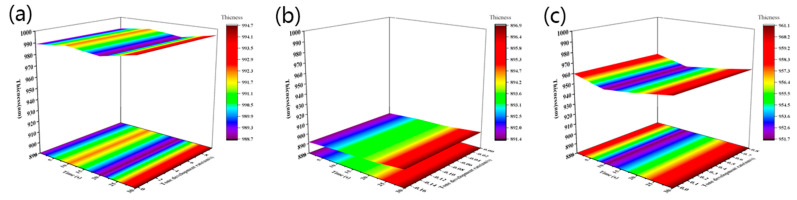
The dissolution characteristics of different BOC concentrations in 2.38 wt% TMAH solution: JB20 (**a**), JB25 (**b**) and JB30 (**c**) at different times of film thickness change and development speed change.

To evaluate the performance of these BOC-protected resins in the resist and to compare them with normal DNQ resists, we used the following resist formulations. Unprotected resin JB480 was mixed with a commercially available PAC (PAC430), which is 2,3,4,4′-tetrahydroxybenzophenone, and DNQ-5 ester (JB-DNQ), at approximately 30 wt% of PAC to JB480 resin. The JB resins were formulated with PAG HG-108 in PGMEA at the proper solid content to form CA resists (PAG is approximately 2 wt% of JB resin). These resist samples were coated on silicon wafers and baked at 110 °C for 5 min. Then, all the resist films were developed with standard 2.38 wt% TMAH developer for different times, and the film loss was measured. The results are summarized in Table 3 and illustrated in Figure 6.

As shown in Table 3, the initial film thickness of JB-DNQ was 1569.1 nm, and the film thickness decreased by 552.8 nm within 30 s in the developing solution. JB20, JB25 and JB30 were all able to control the film thickness loss value within 10 nm within 30 s. Therefore, the JB-DNQ resist had a high dissolution rate in TMAH compared to other JB20, JB25 and JB30 resists and was weak to longer developer times. The developer resistance was not enough to protect the film. However, all of the resist samples of BOC-protected resins had enough TMAH resistance. JB25 with a 25 mol% BOC protection ratio showed almost no film loss. The BOC ratios of the 20 mol% and 30 mol% samples fluctuated.

The film loss of JB-DNQ samples at 10 s, 20 s and 30 s of TMAH development showed linear film thickness loss due to the inhibition effect of the resin and DNQ combination. This result has no significant relationship with DNQ anti-alkaline. However, the BOC-protected JB resin showed stable developer resistance. The JB20, JB25 and JB30 resists were stable with very little film loss. The film thickness of JB25 was more stable in the other three samples. All these results were estimated to be the combination effects of GMA and BOC modification. From this point of view, we concluded that JB25 with a 25 mol% BOC ratio had balanced effects.

**Figure 7:**
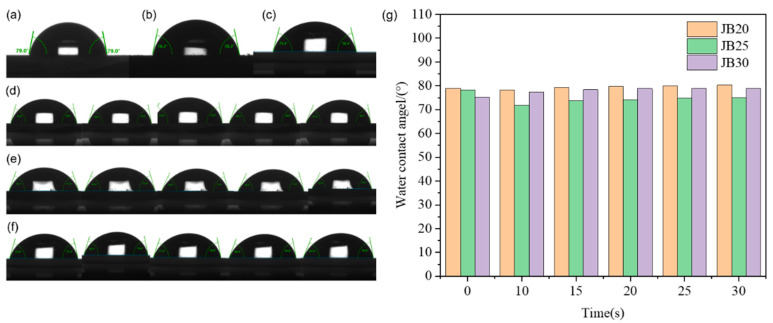
Contact angle test diagram of JB20, JB25 and JB30: (**a**–**g**) the static contact angles of JB20 (**a**), JB25 (**b**) and JB30 (**c**) and the dynamic contact angles of JB20 (**d**), JB25 (**e**) and JB30 (**f**) at 10°, 15°, 20°, 25° and 30°, respectively.

Figure 7 shows the static contact angle and rolling contact angle of JB20, JB25 and JB30, where the rolling contact angle is used to test the wettability of the JB series surface and water under an angle change of 0°–30°. The changes under the five angles of 10°, 15°, 20°, 25° and 30° were selected for a comparison, and it is found that the static contact angles of JB20, JB25 and JB30 have minor differences, and the static contact angles of JB20 and JB25 are larger than that of JB30. Meanwhile, the dynamic contact angle of JB25 is the smallest among the three, 75° and 63° for the forward and backward angles, respectively. Comparison shows that JB25 has the best hydrophobic performance. The hydrophobicity is due to the introduction of the BOC group, which protects the resin in the resist material, and the penetration of hydrophilic TMAH can also be considered to be partially prevented during the development process, so compared with JB25 and JB30, a higher film retention rate and lower dissolution rate of JB25 can be obtained, which corresponds to the results in Table 1. Since JB20, JB25 and JB30 are high molecular weight polymers themselves, the high molecular weight resist has different degrees of hydrophobic strength, the number of BOC groups required for dissolution during the reaction to protect the reaction is higher and the high protection ratio leads to an increase in the dissolution rate. The interaction between different photoactivities leads to inhibition of the reaction dissolution between the light-sensitive agent and the phenolic resin, and the induction of the deprotonation of phenolic hydroxyl groups in the phenolic resin in TMAH accelerates the dissolution of the resist. The increase in BOC content does not produce the same trend of dissolution properties, which leads to different hydrophobic properties of JB20, JB25 and JB30.

**Figure 8:**
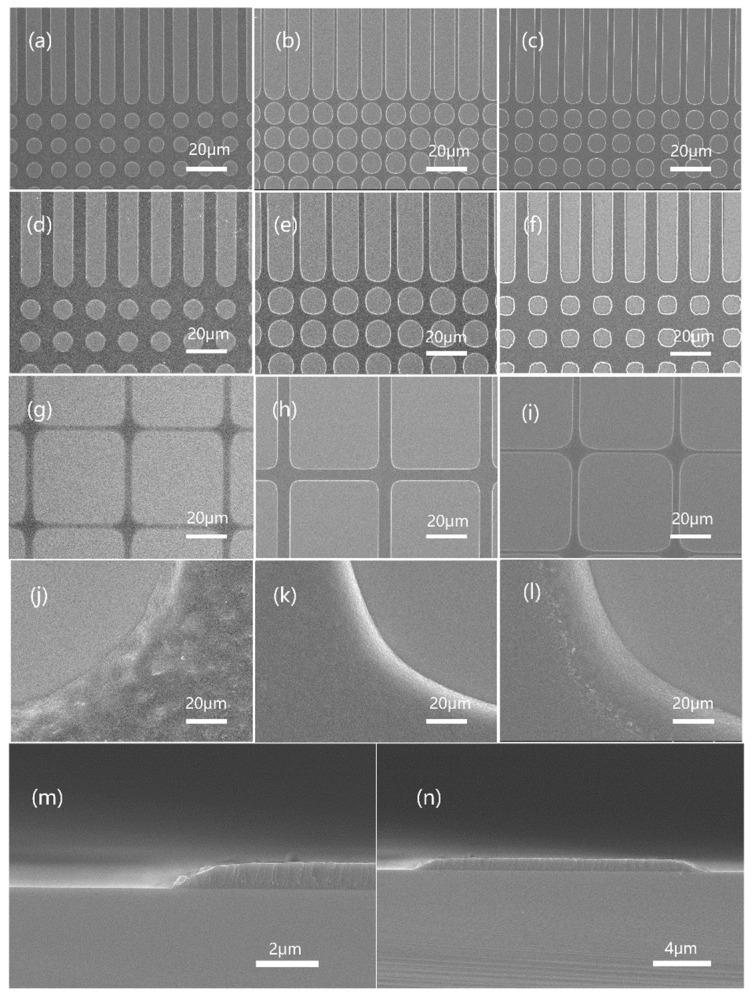
Topography of JB20, JB25 and JB30: JB20 patterned with 6 μm (**a**), 8 μm (**d**) and 10 μm (**g**) masks. JB25 patterned with 6 μm (**b**), 8 μm (**e**) and 10 μm (**h**) masks. JB30 patterned with 6 μm (**c**), 8 μm (**f**) and 10 μm (**i**) masks. Local enlargement of JB20 (**j**), JB25 (**k**) and JB30 (**l**) morphology. Cross-sectional morphology of JB25 under the mask of 16 μm (**m**,**n**).

The resist pattern developed on the silicon wafer from contact exposure with an analog chrome mask was observed with a scanning electron microscope. The SEM images of JB resists with different BOC ratios are shown in Figure 8. The brighter area in the picture is silicon substrate (on which the resist was developed), and the darker area is the remaining resist film. Mask patterns of 4 um, 6 um, and 10 um of JB20, JB25 and JB30 (trench and hole) showed that JB25 gave a clear image and higher photospeed. The surface pictures of each sample’s 10 um pattern corner (j, k, l) showed that JB20 (j) had some roughness owing to its lower developer resistance, while the other two samples had smooth surfaces or better TMAH resistance. The results showed that JB25 with a BOC ratio of 25 mol% was the best among the three samples.

**Figure 9:**
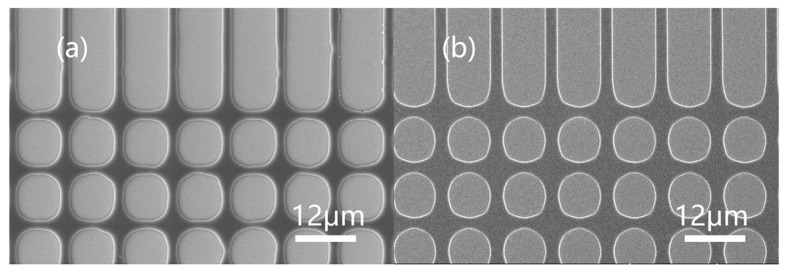
Topography of 12XT and JB25: 12XT patterned under 8 μm (**a**) mask. JB25 patterned under 8 μm (**b**) mask.

To better compare the lithographic performance of JB25, we used a commercial CA-type AZ-12XT photoresist (by Merck AG, Tokyo, Japan), which is based on protected p-hydroxystyrene resin (PHS). AZ-12XT has mature application in i-line lithography with high sensitivity and high resolution compared to the normal DNQ resist. However, its cost is much higher than that of a normal Novolak/DNQ resist. The same lithographic process conditions were applied to AZ-12XT and JB25 with similar thicknesses, prebaking (120 °C for 5 min) and postexposure baking (110 °C for 3 min, exposure energy approximately 190 mJ/cm^2^). It can be seen from Figure 9 that both AZ-12XT and JB25 showed clear patterns, which proved that the JB25 photoresist could be effectively applied to the g-line and i-line to replace the costly PHS-based CA photoresist.

## 4. Conclusions

The preparation of a synthetic resin material with good photolithographic properties requires not only considering photolithography as a process chain but also the synthesis, characterization and formulation of the photographic resin. The synthetic properties of resins for photoresists are considered in two aspects: on the one hand, on a microscopic level, the specific chemical and mechanical properties involved in the synthesis and characterization of the resin, and on the other hand, on a macroscopic level, by mixing other compounds to modify the properties of synthetic resins for photoresists to obtain the final formulation. This work achieves a certain degree of validation in both aspects. The structure and morphological characteristics of the synthesized JB series resins were characterized microscopically using UV spectroscopy, IR spectroscopy, GPC and resist formulations. An appropriate ratio of 25 mol% BOC was obtained by adding GMA and optimizing the BOC content to achieve the best formulation of synthetic resins for photoresists on a macroscopic scale. Using contact exposure, JB20, JB25 and JB30 photoresist resin materials with excellent resolution were obtained, and it was observed that JB20, JB25 and JB30 photoresists all had clear positive development images from photoresist films exposed at 405 nm under UV light with an exposure dose of approximately 190 mJ/cm^2^. The resist with JB25 resin had a higher resolution and fine positive image of clear lines and spatial patterns, so the content of BOC has an important influence on photoresist pattern development, and the ratio of 25 mol% BOC addition is the key regulation point of the resin for JB series photoresists. Comparison with AZ-12XT showed the potential to replace PHS-based resists. In conclusion, we have developed a synthetic route for a modified phenolic resin for CA-type g-line and i-line photoresist by the addition of GMA to the resin followed by BOC protection. This opened a new way for a stable supply of i-line and g-line CA resists with a cost advantage and broad selection of resins, also indicating the practical social application value and great potential of the modified phenolic resin to replace the expensive PHS resin in the photoresist field and its lithography process.

## Figures and Tables

**Table 1:** Wide distribution unknown sample universal peak table.

Samples	$M_{n}\;(\mathrm{Daltons})$	$M_{w}\;(\mathrm{Daltons})$	$M_{p}\;(\mathrm{Daltons})$	Polydispersity
JB480	1875	7868	4771	4.20
JB20	2070	6504	4792	3.14
JB25	2080	6510	4771	3.13
JB30	2140	6803	4792	3.18

$M_{n}$ is number average molecular weight, $M_{w}$ is weight average molecular weight, $M_{p}$ is peak molecular weight.

**Table 2:** GMA-modified phenolic resin.

GMA-Modified Phenolic Resin							
initial thickness (nm)	773.5	773.5	882.6	719.7	820.7	731.4	531
ratio of GMA (mol%)	0%	5%	10%	15%	20%	25%	30%
film thickness loss at 20 s (nm)	644.9	644.9	585.1	537.4	454.3	292.6	20.3

**Table 3:** Sample film thickness loss.

Samples	JB20 (nm)	JB25 (nm)	JB30 (nm)	JB-DNQ (nm)
initial thickness	988.9	891.4	959.8	1569.1
film thickness loss at 10 s	6.6	0	8.1	79.9
film thickness loss at 20 s	0.2	0	4.1	279.7
film thickness loss at 30 s	4.2	0	1.3	552.8

## Data Availability

The data presented in this study are available on request from the corresponding author.
